# Identification of key genes in osteoarthritis development: biomarker discovery and therapeutic targets

**DOI:** 10.3389/fmed.2025.1518580

**Published:** 2025-07-02

**Authors:** Liming Wu, Disheng Wen, Weizhou Wang, Yanghao Wang, Li Zhang

**Affiliations:** ^1^Department of Orthopedics, Sichuan Santai County People's Hospital, Mianyang, China; ^2^Department of Orthopedics (Fourth), Dali Bai Autonomous Prefecture People's Hospital, Dali, China; ^3^Department of Orthopedics, The First Affiliated Hospital of Kunming Medical University, Kunming, China; ^4^Department of Pathology, The First Affiliated Hospital of Kunming Medical University, Kunming, China

**Keywords:** osteoarthritis, biomarkers, bioinformatics, diagnostic markers, drug prediction

## Abstract

**Introduction:**

Osteoarthritis (OA) is the most common joint disorder and a leading cause of disability in the older adult. Early diagnosis and treatment are crucial for effective disease management and improved outcomes. This study aims to identify key genes involved in OA progression using bioinformatics, which may serve as diagnostic biomarkers and therapeutic targets.

**Methods:**

Synovial tissue sequencing data (GSE1919, GSE55235, GSE82107) were retrieved from the Gene Expression Omnibus (GEO) database. Differentially expressed genes (DEGs) were analyzed using Gene Ontology (GO), Kyoto Encyclopedia of Genes and Genomes (KEGG), and protein–protein interaction (PPI) network analysis. ROC curve analysis was used to assess diagnostic potential, and results were validated using the GSE29746 dataset and synovial tissues from five OA patients and controls.

**Results:**

A total of 33 common DEGs were identified across three datasets. Four hub genes (CXCL8, CXCL2, DUSP5, TNFSF11) showed high diagnostic potential [area under the receiver operating characteristic curve (AUC) > 0.8]. These genes were also linked to potential therapeutic agents, including lipopolysaccharide and acetaminophen.

**Conclusion:**

CXCL8, CXCL2, DUSP5, and TNFSF11 represent novel multi-functional biomarkers that advance OA research by addressing two critical limitations of prior biomarker studies: (1) overcoming the diagnostic inadequacy of single-biomarker approaches through synergistic clusters, and (2) revealing an unreported integrative mechanism linking inflammatory pathways (CXCL8/2) and bone remodeling processes (TNFSF11/DUSP5). This dual diagnostic-therapeutic potential significantly expands the clinical applicability of OA biomarkers.

## 1 Introduction

Osteoarthritis (OA) is a common age-related degenerative disease that results in chronic joint pain and functional impairment, severely affecting patients’ quality of life (1, 2). Current therapeutic approaches predominantly target symptom relief, particularly inflammation and pain management, but are largely ineffective at preventing disease progression (3). As a result, many patients with end-stage OA ultimately require surgical intervention (4, 5). Early diagnosis and intervention are critical for improving patient outcomes and optimizing healthcare resource allocation. OA is marked by synovial inflammation, subchondral bone remodeling, and cartilage degradation, with synovial lesions increasingly recognized as central to disease development and joint pain (6–8). Synovial changes alter the intra-articular environment, playing a key role in the breakdown of the extracellular matrix and cartilage degradation (9, 10). Understanding the role of synovial tissue lesions in OA progression is thus of paramount importance (11).

While previous biomarker studies in OA have identified individual candidates (e.g., COMP, CTX-II), they are limited by a focus on singular targets that fail to capture the multifactorial pathogenesis of OA. This study addresses this critical gap by identifying functionally interconnected biomarker clusters that collectively reflect OA’s complexity. We establish three key methodological advances: (1) Integrated analysis of four independent synovial tissue datasets (training: GSE1919, GSE55235, GSE82107; validation: GSE29746) enhances the reliability of DEG identification, (2) Combined application of GO, KEGG, GSEA, and PPI network analysis enables systematic elucidation of both functional and interactive properties of hub genes, (3) Multi-level validation strategy incorporating ROC analysis, independent dataset replication, and patient-derived tissue verification. This approach uniquely identifies CXCL8, CXCL2, DUSP5, and TNFSF11 as a novel biomarker cluster—simultaneously implicating inflammatory pathways (CXCL8/2) and bone remodeling processes (TNFSF11/DUSP5)—providing a framework for combined diagnostic panels and multi-target therapies that transcend conventional single-biomarker limitations. In this study, we analyzed synovial tissue gene expression profiles from four datasets obtained from the Gene Expression Omnibus (GEO) database, including data from OA patients and healthy controls, which were divided into training and validation sets. Bioinformatics analyses, including Gene Ontology (GO) enrichment, Kyoto Encyclopedia of Genes and Genomes (KEGG) pathway analysis, Gene Set Enrichment Analysis (GSEA), and Protein–Protein Interaction (PPI) network analysis, were employed to identify differentially expressed genes (DEGs) and to elucidate their biological functions. The diagnostic accuracy of hub genes was assessed using receiver operating characteristic (ROC) analysis, which also facilitated predictions regarding OA progression and early therapeutic strategies based on these diagnostic genes.

This comprehensive approach offers new insights into the regulatory genes and molecular mechanisms underlying OA, contributing to a more detailed understanding of the disease’s pathophysiology and potential therapeutic avenues. While specific clinical biomarkers for OA remain elusive, *our identification of functionally interconnected biomarker clusters (CXCL8, CXCL2, DUSP5, TNFSF11) provides a novel framework for developing combined diagnostic panels and multi-target therapies, surpassing the limitations of conventional single-biomarker approaches.

## 2 Materials and methods

### 2.1 Data collection

**Figure 1:**
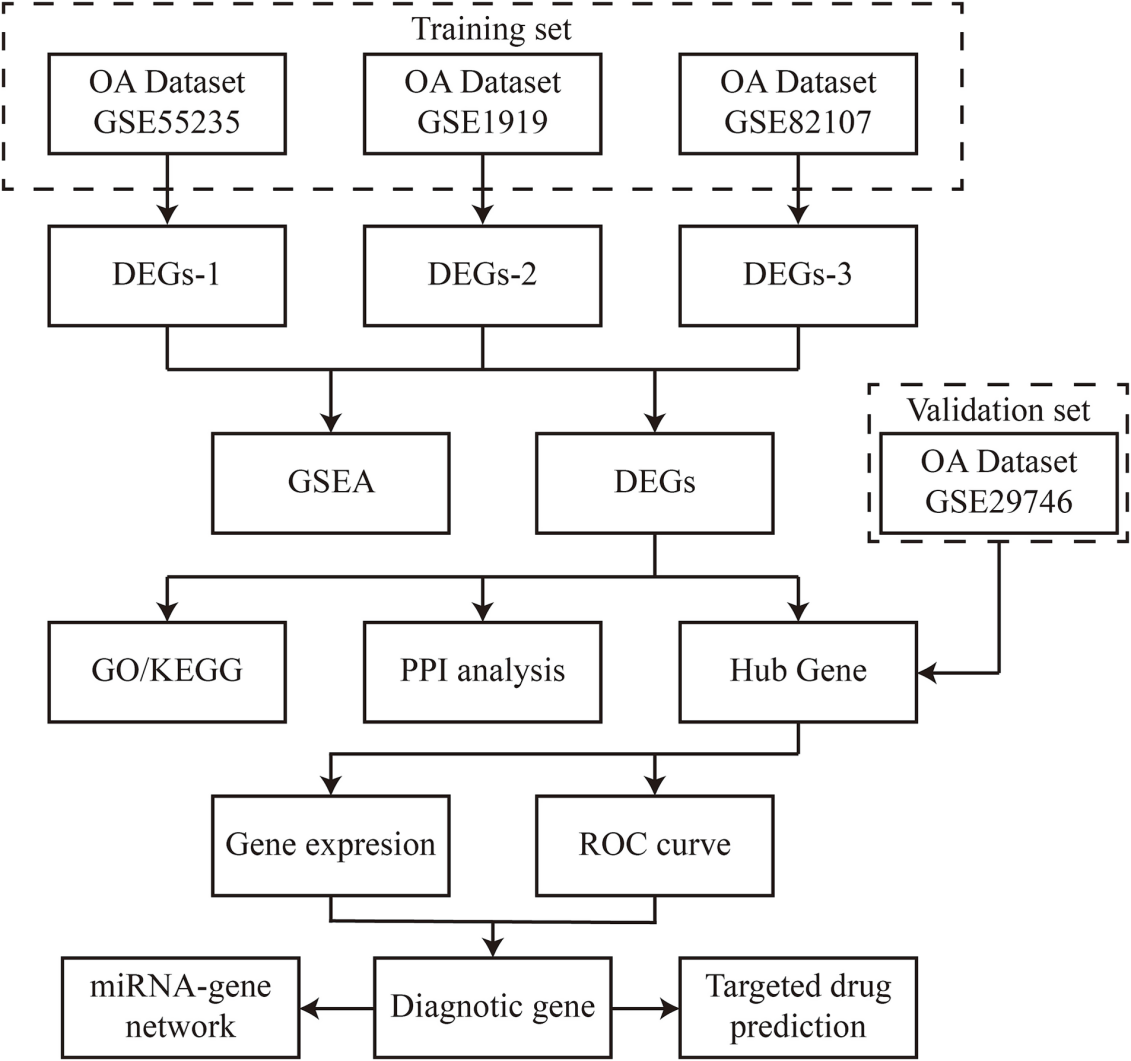
Flowchart presentation of this study.

The gene expression values from datasets GSE1919, GSE55235, GSE82107, and GSE29746 were retrieved from the Gene Expression Omnibus (GEO) database using the GEOquery package. Synovial biopsy samples from both OA patients and healthy controls were used to identify differentially expressed genes (DEGs) associated with OA, potentially serving as disease-specific signature genes. To ensure analytical rigor, the four datasets were divided into training sets (GSE1919, GSE55235, GSE82107) and an independent validation set (GSE29746) based on sample size (>20 samples per group) and platform compatibility (all Affymetrix platforms). The data were normalized using the “normalizeBetweenArrays” function in the limma [3.52.2] package in R (version 4.2.1). Sample subgroup clustering was visualized through principal component analysis (PCA) plots. Figure 1 provides a flowchart outlining the study design.

### 2.2 Analysis of differentially expressed genes

Differential gene expression analysis between OA and control groups was conducted using the limma [3.52.2] package in R (version 4.2.1). The thresholds |log2(FC)| > 1 and *p*-value < 0.05 were selected based on: (1) Biological significance: |log2(FC)| > 1 ensures ≥2-fold expression changes, a standard cutoff for functionally relevant alterations in synovial transcriptomics; (2) Statistical rigor: *p*-value < 0.05 with Benjamini-Hochberg correction controls false discovery rates (FDR) in multi-dataset analyses (12). The significant DEGs were visualized using heatmaps (ComplexHeatmap [2.13.1]) and volcano plots (ggplot2 [3.3.6]). Gene Set Enrichment Analysis (GSEA) was performed with clusterProfiler (reference gene set: c2.cp.all.v2022.1. Hs.symbols.gmt). Enriched pathways were filtered at p.adj < 0.05 and FDR (*q*-value) < 0.25.

### 2.3 Expression analysis of common DEGs and hub genes

The intersection of DEGs from the three training sets was visualized using ggplot2 [3.3.6] and VennDiagram [1.7.3] in R (version 4.2.1). Subsequently, Gene Ontology (GO) and KEGG enrichment analyses were conducted using the clusterProfiler [4.4.4] package, with input gene IDs transformed using org. Hs.eg.dbR. Protein–protein interaction (PPI) network analysis was performed via the STRING database (version 11.5) with the following parameters: Minimum required interaction score: 0.7 (high confidence), Active interaction sources: Experiments & Databases, Excluded disconnected nodes. The resultant network (contained 33 nodes and 87 edges) was imported into Cytoscape software (version 3.9.0). Hub genes were identified using the Edge Percolated Component (EPC) algorithm in Cytohubba plugin with default parameters (edge weight: combined score; node score: betweenness centrality). The top 10 hub genes were selected based on descending EPC scores. The expression levels and Spearman correlations of DEGs were analyzed across the three training sets.

### 2.4 Identification of diagnostic genes

We illustrated the expression levels of key genes between OA patients and healthy controls using scatter plots and box plots. ROC analysis was conducted using the pROC [1.18.0] package in R (version 4.2.1), with results visualized using ggplot2 [3.3.6]. Training phase: Internal validation via 10-fold cross-validation repeated 3 times on combined training sets (GSE1919 + GSE55235 + GSE82107), Validation phase: Independent testing on GSE29746 dataset. Diagnostic genes were selected from both the training and validation datasets using an AUC threshold of >0.800.

### 2.5 Synovial sample collection and validation for differential expression

Validation cohort selection criteria: Synovial tissues from five OA patients (Kellgren-Lawrence grade II-III) and five age/sex-matched healthy controls (mean age 58.2 ± 4.3 vs. 56.8 ± 5.1 years, *p* > 0.05) were collected at our hospital. Inclusion criteria for OA patients: (1) Diagnosed according to ACR clinical criteria, (2) No history of inflammatory arthritis or joint infection, (3) No intra-articular injections within 6 months. Control group criteria: Individuals undergoing knee arthroscopy for meniscal injuries without radiographic OA (Kellgren-Lawrence grade 0) or synovitis (histological evaluation). Informed consent was obtained from all participants, and the study was approved by the hospital’s ethics committee. RNA isolation and extraction were performed using the TRIzol method as per the manufacturer’s protocol (Invitrogen). Complementary DNA (cDNA) synthesis was performed using a reverse transcription kit (Vazyme, 7E581J1). To ensure reproducibility, all validation experiments included: (1) Technical triplicates for each sample, (2) Negative controls without template, (3) Reference gene normalization (GAPDH and ACTB). RNA quality was assessed by: Nanodrop 2000 spectrophotometer (Thermo Fisher): OD260/280 ratios 1.9–2.1. For gene amplification, 1 μL of cDNA template and 0.6 μL of primers per gene were used in a 20-μL reaction. PCR amplification included an initial denaturation at 95°C for 10 min, followed by 40 cycles of denaturation at 95°C for 10 s, annealing, and extension at 60°C for 32 s.

### 2.6 Construction of miRNA-gene regulatory network

The starBase database^1^ was used to predict the interactions between diagnostic genes and miRNAs. The resulting miRNA-gene regulatory network was visualized using Cytoscape software (version 3.9.0).

### 2.7 Prediction of potential therapeutic drugs

Potential therapeutic drugs for OA were predicted by constructing gene-drug interaction networks using the Comparative Toxicogenomics Database (CTD). The CTD integrates data from published studies on chemical compounds, genetics, phenotypes, and diseases, aiding in the prediction of potential drug targets for key OA-related genes. Drug Prediction Protocol: Gene-drug interaction retrieval: Input: Official gene symbols of diagnostic markers, Filters: Interaction score >30, evidence count ≥5. Therapeutic relevance scoring: Mechanism score: 0–1 (1 = direct OA pathway evidence), Clinical score: 0–1 (1 = Phase III trial/OA approval), Priority score = 0.6 × Mechanism + 0.4 × Clinical. Manual curation: Excluded non-human studies: Removed compounds with off-target toxicity (LD50 < 50 mg/kg).

## 3 Results

### 3.1 Identification of DEGs in synovial tissues of OA patients and functional analysis

**Figure 2:**
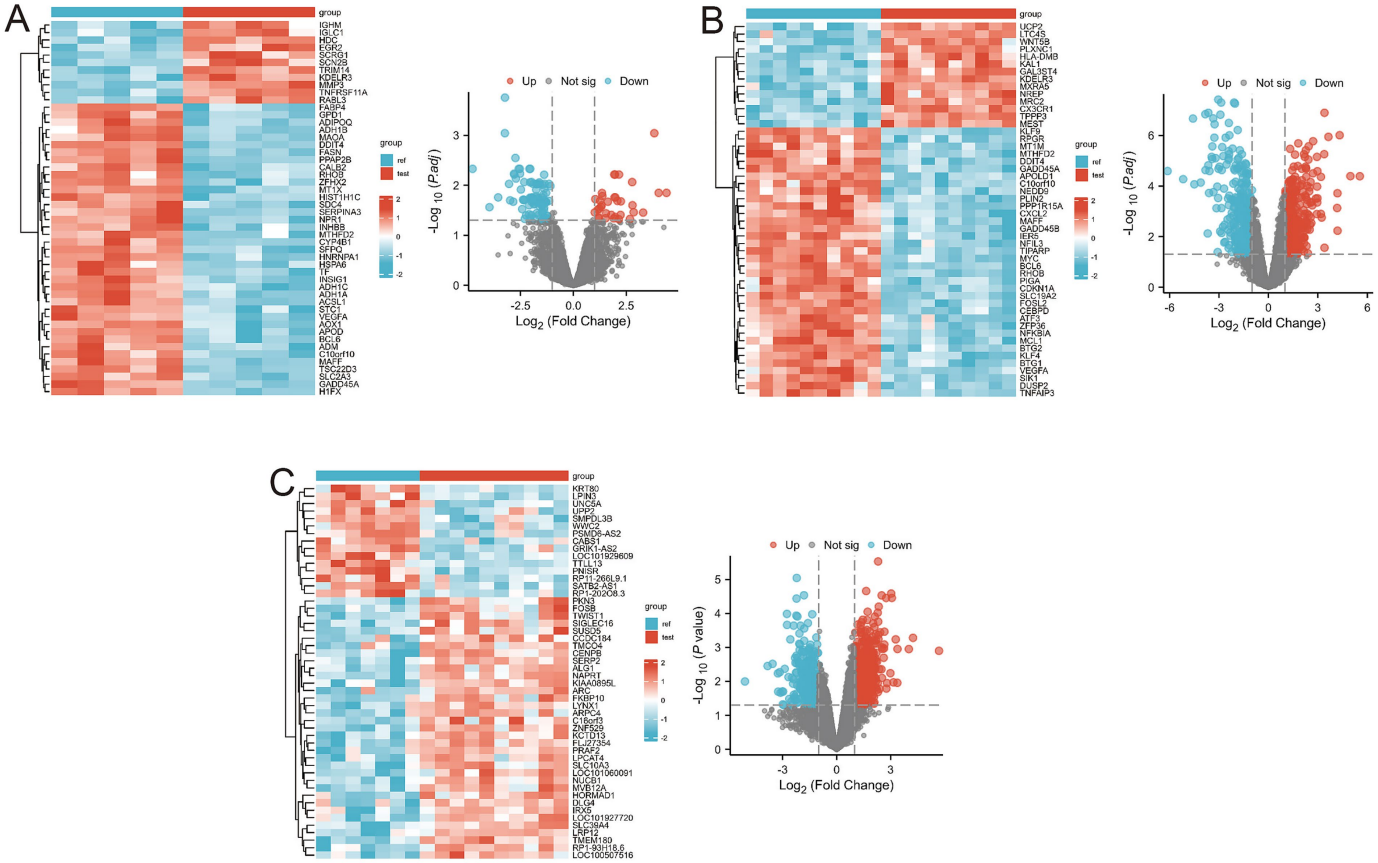
Differential gene expression in the three training sets. **(A)** Volcano and heatmap display of differential gene expression results for the GSE1919 dataset; **(B)** Volcano and heatmap display of differential gene expression results for the GSE55235 dataset; **(C)** Volcano and heatmap display of differential gene expression results for the GSE82107 dataset.

Using the thresholds of |log2(FC)| > 1 and *p*-value < 0.05, a total of 541 differentially expressed genes (DEGs) were identified from the GSE1919 training set, with 240 significantly upregulated and 301 significantly downregulated genes (Supplementary Table 1). DEGs were visualized using volcano plots and heat maps (Figure 2A). Similarly, 958 DEGs were identified from the GSE55235 training set, including 519 significantly upregulated and 439 significantly downregulated genes (Supplementary Table 2), visualized through volcano plots and heat maps (Figure 2B). In the GSE82107 training set, 1,213 DEGs were identified, comprising 728 significantly upregulated and 485 significantly downregulated genes (Supplementary Table 3), also visualized using volcano plots and heat maps (Figure 2C).

**Figure 3:**
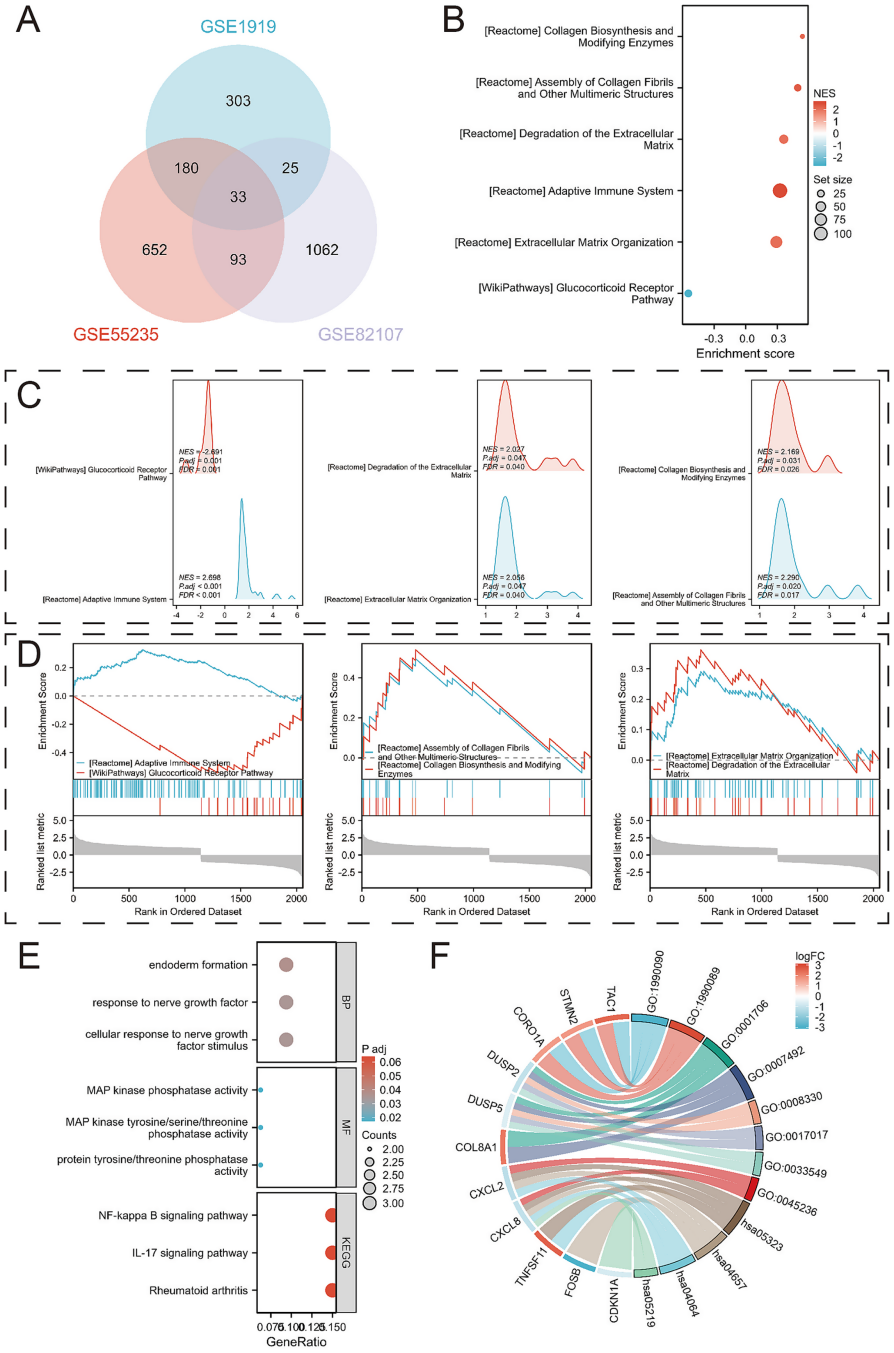
Identification of DEGs and their functional analysis. **(A)** Venn diagram between overlapping genes; **(B)** Bubble diagram results of enrichment analysis using Gene Set Enrichment Analysis (GSEA); **(C)** Mountain range diagram results of enrichment analysis using GSEA; **(D)** Classical results of enrichment analysis using GSEA; **(E)** Bubble diagrams of GO and KEGG pathways enriched for 33 DEGs; **(F)** Chord diagrams of GO and KEGG pathways enriched for 33 DEGs.

Gene Set Enrichment Analysis (GSEA) revealed several key pathways associated with OA progression, including REACTOME_ADAPTIVE_IMMUNE_SYSTEM, WP_GLUCOCORTICOID_RECEPTOR_PATHWAY, REACTOME_ASSEMBLY_OF_COLLAGEN_FIBRILS_AND_OTHER_MULTIMERIC_STRUCTURES, REACTOME_COLLAGEN_BIOSYNTHESIS_AND_MODIFYING_ENZYMES, REACTOME_EXTRACELLULAR_MATRIX_ORGANIZATION, and REACTOME_DEGRADATION_OF_THE_EXTRACELLULAR_MATRIX (Figures 3B–D; Supplementary Table 4). By integrating DEGs from all three training sets, we identified 33 overlapping DEGs (Figure 3A).

Further analysis of these 33 overlapping DEGs revealed enriched biological processes (BPs) such as cellular response to nerve growth factor stimulus, response to nerve growth factor, and endoderm formation. In terms of molecular function (MF), the DEGs were enriched for protein tyrosine/threonine phosphatase activity, MAP kinase tyrosine/serine/threonine phosphatase activity, and MAP kinase phosphatase activity. KEGG analysis linked the DEGs to pathways including Rheumatoid arthritis, IL-17 signaling pathway, and NF-kappa B signaling pathway (Figures 3E–F; Supplementary Tables 5, 6).

### 3.2 PPI network analysis and hub gene identification

**Figure 4:**
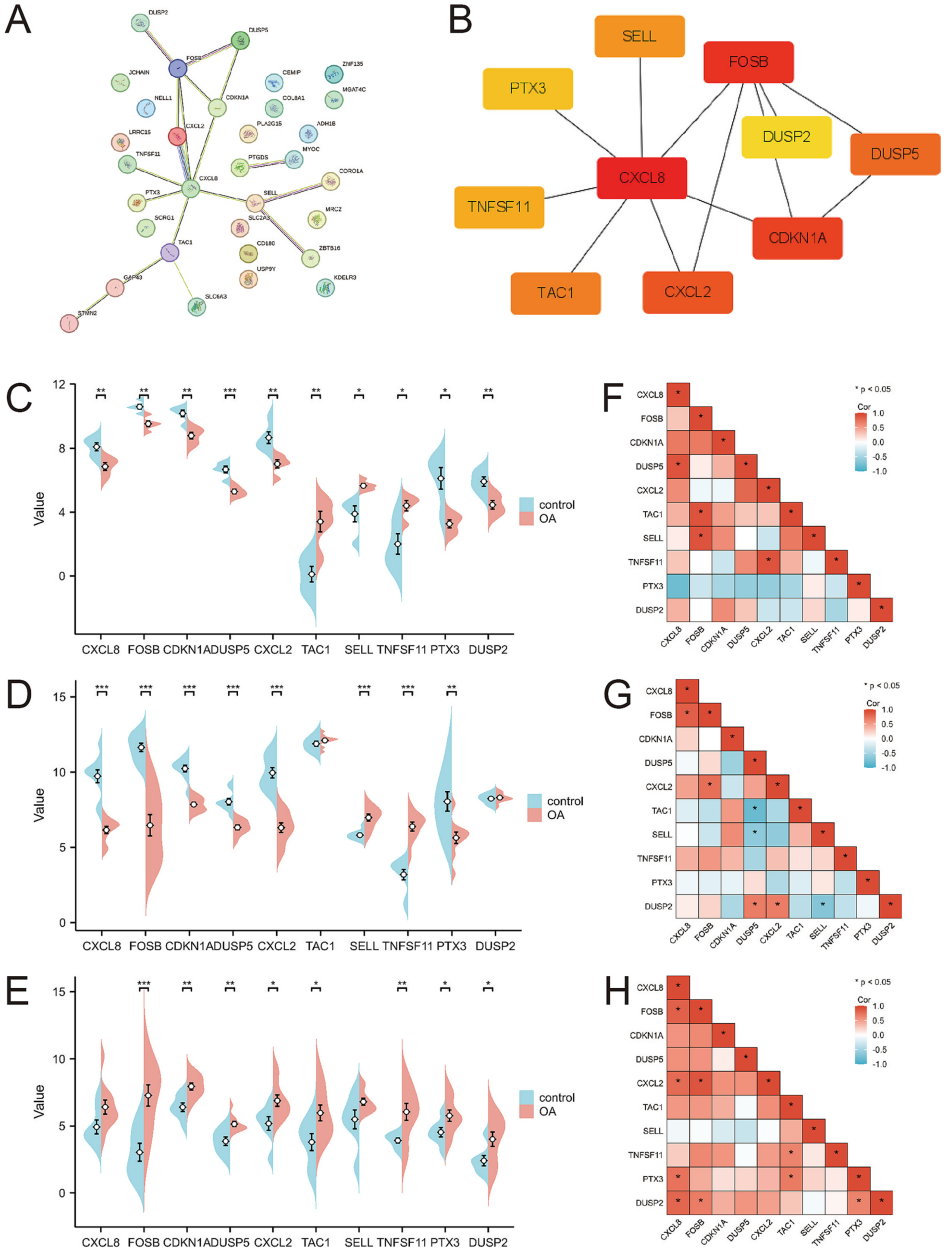
Protein–protein interaction network and expression of hub genes. **(A)** Protein–protein interaction network constituted by DEGs; **(B)** Top 10 hub genes; **(C–E)** Pod plots of the expression of the 10 hub genes in GSE1919, GSE55235, and GSE82107. **(F–H)** Spearman correlation of the 10 hub genes in GSE1919, GSE55235, and GSE82107.

**Figure 5:**
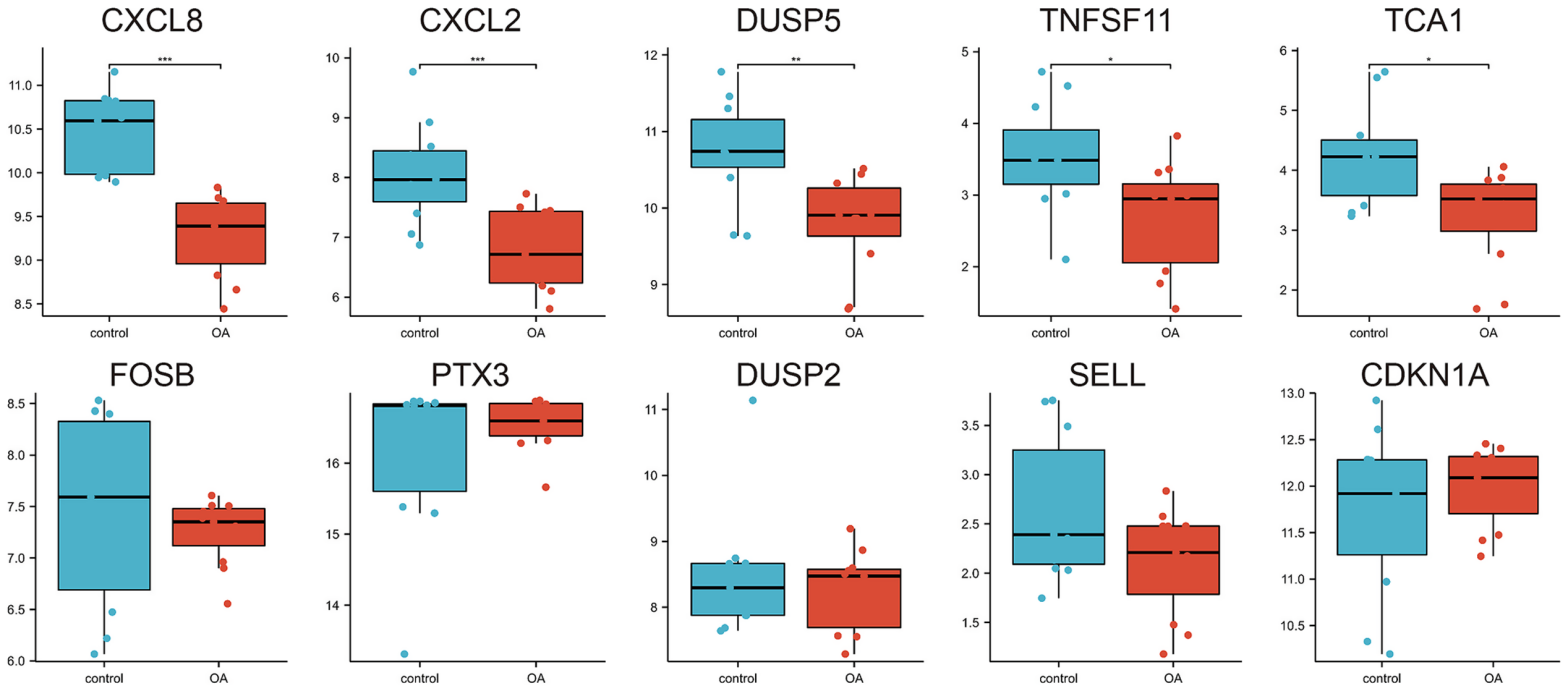
Expression of the 10 hub genes in the GSE29746 validation set.

A protein–protein interaction (PPI) network comprising the 33 differentially expressed genes was constructed using the STRING database to elucidate their interactions (Figure 4A). The top 10 hub genes were identified using the Cytohubba plugin in Cytoscape software (Figure 4B). The expression levels of these hub genes across the three training sets are shown in Figures 4C–E, and their correlations are presented in Figures 4F–H. Significant expression differences were observed for CXCL8, CXCL2, DUSP5, TNFSF11, and TCA1 in the GSE29746 validation set (Figure 5).

### 3.3 Identification and validation of diagnostic signature biomarkers

**Figure 6:**
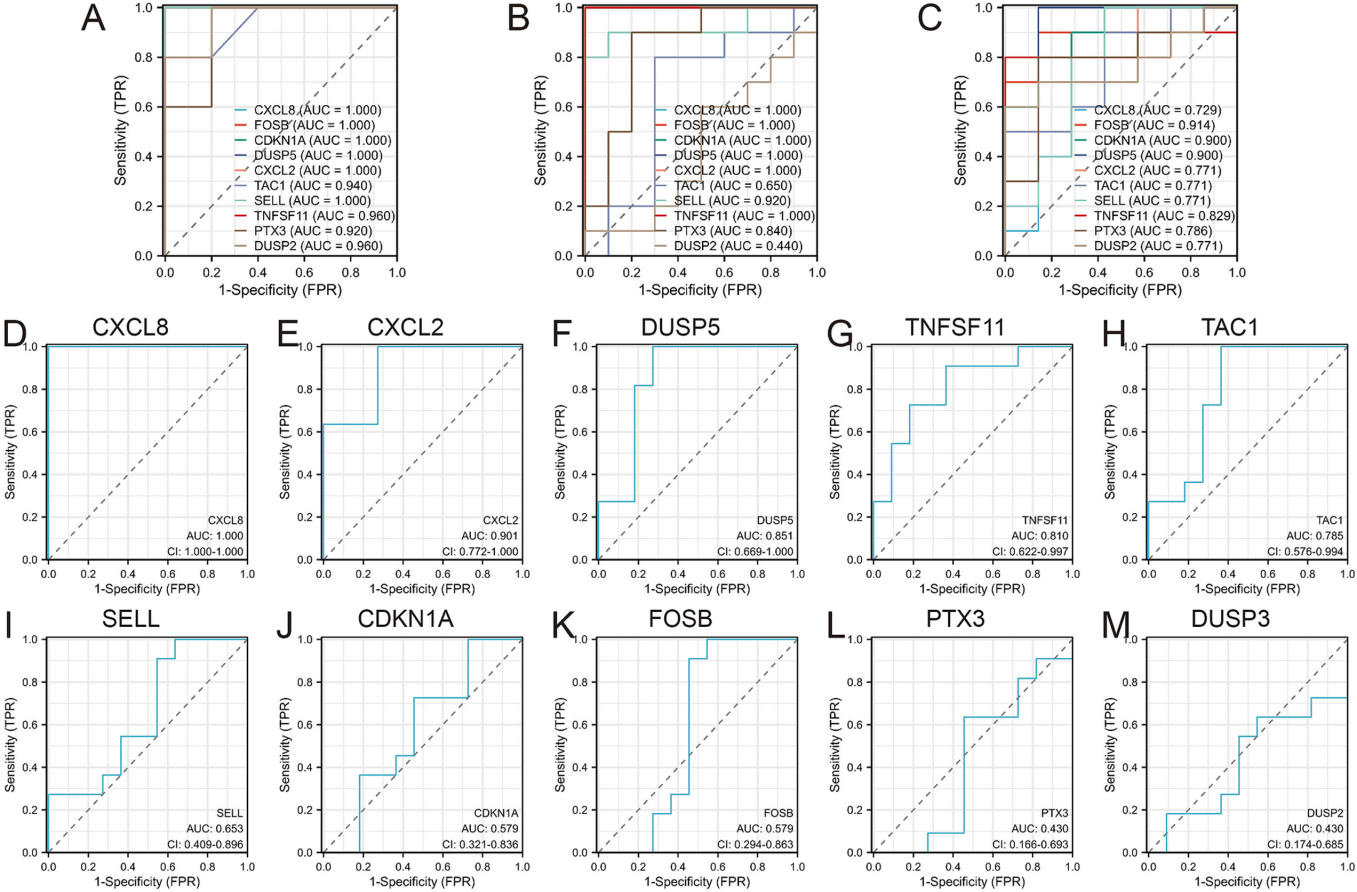
Diagnostic values of the 10 Hub genes. **(A)** Diagnostic values of 10 hub genes in GSE1919 training set; **(B)** Diagnostic values of 10 hub genes in GSE55235 training set; **(C)** Diagnostic values of 10 hub genes in GSE82107 training set; **(D–M)** Diagnostic values of 10 hub genes in GSE29746 validation set.

Receiver operating characteristic (ROC) curves were employed to assess the diagnostic value of the 10 hub genes in OA (Figures 6A–C). The results indicated that, with the exception of DUSP2, the remaining nine genes showed high diagnostic value (AUC > 0.7) for OA diagnosis. Validation in the GSE29746 set (Figures 6D–M) confirmed that CXCL8 [AUC = 1.000 (95% CI 1.000–1.000)] and CXCL2 [AUC = 0.901 (95% CI 0.722–1.000)] had excellent predictive accuracy for OA onset (AUC > 0.9), while DUSP5 [AUC = 0.851 (95% CI 0.669–1.000)], TNFSF11 [AUC = 0.810 (95% CI 0.622–0.997)], and TAC1 [AUC = 0.785 (95% CI 0.576–0.884)] displayed high accuracy (AUC > 0.7).

**Figure 7:**
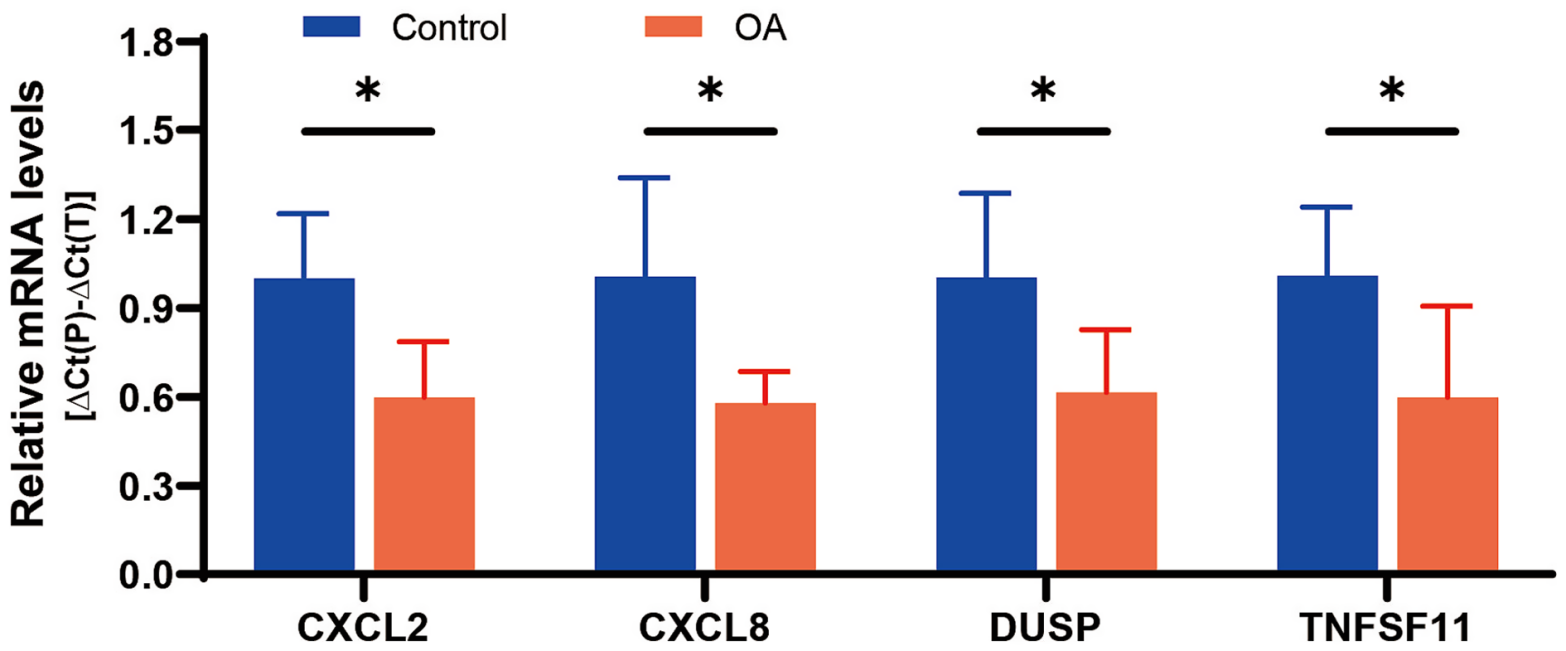
mRNA expression of 4 diagnostic genes in tissues.

Ultimately, four diagnostic genes with AUC > 0.8 (CXCL8, CXCL2, DUSP5, and TNFSF11) were identified. Synovial tissues from five pairs of OA patients and healthy controls were collected to validate these genes. PCR experiments revealed significant differential expression of these four genes in OA synovial tissues: CXCL2 (*p* = 0.0140), CXCL8 (*p* = 0.0255), DUSP5 (*p* = 0.0401), and TNFSF11 (*p* = 0.0444), supporting their diagnostic relevance in OA (Figure 7).

### 3.4 Construction of miRNA-gene networks and prediction of potential therapeutic drugs

**Figure 8:**
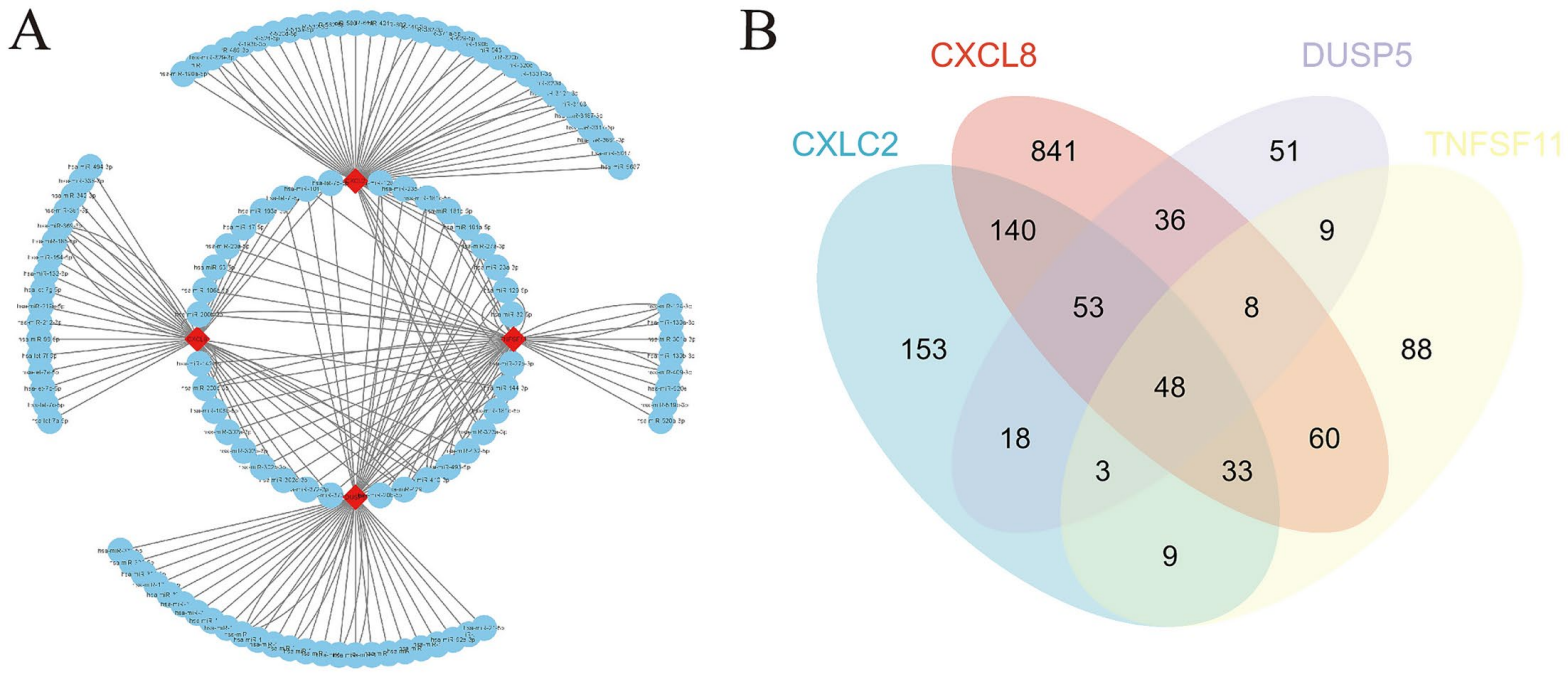
miRNA gene network and potential therapeutic drug prediction. **(A)** Co-expression network of diagnostic genes and target miRNAs; **(B)** Venn diagram between overlapping potential therapeutic drugs.

The starBase database was used to predict microRNAs (miRNAs) interacting with the four diagnostic genes (CXCL8, CXCL2, DUSP5, and TNFSF11). The top 50 predicted miRNAs for each gene were selected to construct gene networks. Analysis of the miRNA-gene network using Cytoscape identified 36 miRNAs regulating two or more diagnostic genes (Figure 8A; Supplementary Table 7).

Furthermore, potential drugs influencing the expression of these diagnostic genes in OA were predicted. CXCL8 was associated with 1,220 interacting drugs, CXCL2 with 458 drugs, DUSP5 with 227 drugs, and TNFSF11 with 259 drugs. By integrating these, we identified 48 overlapping drugs as potential treatments for OA. These included lipopolysaccharides, acetaminophen, particulate matter, tetrachlorodibenzodioxin, and dexamethasone, among others (Figure 8B; Supplementary Table 8).

## 4 Discussion

OA stands as a primary contributor to chronic pain and physical limitations among older individuals. Its prevalence escalates notably in developed nations with aging populations, thereby posing significant economic and health burdens (13). Despite its widespread occurrence, effective treatment options remain limited (14). Current therapies primarily focus on symptom relief rather than addressing joint damage or halting disease progression, which is critical for restoring normal function (15). Furthermore, the progression and severity of OA are aggravated by various risk factors, including joint injury, repetitive strain, obesity, and genetic predisposition (16). As a result, early diagnosis and targeted treatment are crucial strategies for managing OA. However, the regulatory factors underlying OA development remain poorly understood. Additionally, the lack of reliable biochemical markers (biomarkers) for assessing OA diagnosis and prognosis necessitates the exploration of synovial tissue biomarkers as potential indicators of disease status and treatment outcomes, complementing traditional imaging and clinical assessments (17).

This study focused on bioinformatics-based screening to identify diagnostic biomarkers linked to OA progression in synovial tissues of clinical patients. Previous studies on OA biomarkers have largely centered on analyzing differentially expressed genes (DEGs) in blood or cartilage tissues. For example, Xue et al. (18) identified circRNAs as potential diagnostic biomarkers in the peripheral blood mononuclear cells of OA patients. Similarly, Da et al. (19) used weighted gene co-expression network analysis to identify and validate hub genes associated with OA in cartilage. Ren et al. (20) employed bioinformatics tools to screen for key genes involved in reversing senescence and heterochromatin instability in chondrocytes. PTX3 can alleviate the formation or deterioration of OA through intervention verification responses (21). However, these studies did not explore the role of synovial tissue lesions in OA progression, which our findings bridge through the identification of CXCL8, CXCL2, DUSP5, and TNFSF11 as synovium-specific biomarkers. The CXCL8/IL-8 axis aligns with but extends prior findings on synovial inflammation in OA (22). Our multi-dataset validation strategy (AUC > 0.8) surpasses the single-dataset approaches used in earlier synovial biomarker studies (23). The dual inflammatory-bone remodeling function of TNFSF11 provides novel insights compared to previous single-pathway biomarkers like COMP.

CXCL8 encodes interleukin 8 (IL-8), a key mediator of the inflammatory response. Altered CXCL8 expression in OA synovium suggests its potential as a target for modulating inflammatory responses in macrophages (24, 25). CXCL2, a chemokine and neutrophil chemoattractant, plays important roles in immune responses and is closely linked to osteogenic processes that influence OA progression (26, 27). DUSP5 is a negative regulator of mitogen-activated protein (MAP) kinase signaling, which affects cell proliferation and differentiation, contributing to OA development (28). TNFSF11, a member of the tumor necrosis factor cytokine family, regulates apoptosis and osteoclast differentiation, both of which influence OA progression (29). Notably, our findings resonate with emerging OA mechanisms: The chemokine-osteoclast axis (CXCL8/2-TNFSF11) mirrors the synovial-bone crosstalk observed in recent single-cell studies (30). DUSP5’s MAPK regulatory role complements the mitochondrial dysfunction mechanisms identified in WGCNA-based OA subtyping (23). GO/KEGG and GSEA analyses demonstrated that these four diagnostic genes play significant roles in immune responses and stromal remodeling associated with OA, supporting their utility as biomarkers for OA diagnosis. The predicted drug associations (e.g., acetaminophen-DUSP5 interaction) align with recent breakthroughs: Acetaminophen’s anti-catabolic effects via MAPK inhibition (31), while lipopolysaccharide’s paradoxical effects warrant dose–response validation in synovial fibroblast models. Moreover, the miRNA co-targeting network overlaps with clinically validated OA targets: hsa-miR-196a (upregulated in our study) correlates with miR-29b-5p-based hydrogel therapies for cartilage regeneration (32).

The four synovial biomarkers identified in this study (CXCL8, CXCL2, DUSP5, and TNFSF11) not only advance diagnostic capabilities but also provide actionable insights for targeted OA therapy. Their distinct roles in synovial pathophysiology align with emerging precision medicine strategies: CXCL8/CXCL2 as inflammatory modulators: Elevated expression of these chemokines highlights synovitis as a therapeutic target. Preclinical studies suggest that CXCR2 antagonists (e.g., navarixin), currently in Phase II trials for rheumatoid arthritis (NCT04000789), could be repurposed for OA patients with predominant inflammatory phenotypes. Synovial fluid analysis in such patients may guide personalized anti-inflammatory interventions. As a negative regulator of MAPK signaling, DUSP5 downregulation in OA synovium correlates with cartilage catabolism. This finding supports the potential of MAPK inhibitors (e.g., trametinib) to mitigate OA progression, particularly in patients with low DUSP5 expression. Combining DUSP5 expression profiling with NSAID regimens could optimize therapeutic efficacy. The association between TNFSF11 overexpression and osteoclast activation suggests synergy with RANKL inhibitors like denosumab. Ongoing clinical trials exploring denosumab in erosive OA (NCT04880629) may benefit from stratifying patients based on synovial TNFSF11 levels. While our biomarkers demonstrate high diagnostic accuracy for OA, distinguishing early OA from rheumatoid arthritis (RA) or traumatic synovitis requires combinatorial approaches: Unlike RA, which shows systemic autoimmunity (e.g., anti-CCP antibodies), OA synovium exhibits unique TNFSF11 elevation unaltered by IL-17 inhibition. A diagnostic algorithm integrating serum anti-CCP testing with synovial TNFSF11 quantification could enhance specificity – a priority for future validation. Transient CXCL8 elevation post-injury contrasts with sustained overexpression in chronic OA. Longitudinal monitoring of CXCL8 dynamics may aid differentiation.

Several studies have highlighted the role of miRNA dysregulation in OA pathogenesis. For instance, miR-223 interacts with NLRP3 mRNA to suppress inflammation and promote chondroprotection, inhibiting OA progression (33). miR-146a and miR-140-5p modulate transcription via DNA methylation, alleviating OA progression (34). The miR-130a/HDAC3/PPAR-*γ* signaling axis is also implicated in regulating OA (35). In our study, we constructed a co-expression network of diagnostic genes and their target miRNAs, suggesting that multiple miRNAs co-targeting CXCL8, CXCL2, DUSP5, and TNFSF11 may represent potential therapeutic targets for OA prevention and treatment. However, this study did not conduct preliminary validation of these miRNA targets, which is a limitation. Future research will focus on validating these targets, with the aim of discovering more effective biomarkers for OA diagnosis and treatment.

Our study began by analyzing multiple microarray datasets to identify common differentially expressed genes, thereby minimizing individual variability. Using these common DEGs, a protein–protein interaction (PPI) network was constructed to screen for hub genes linked to the disease. The identified hub genes were then used to generate diagnostic curves and assess differential expression across additional datasets and clinical samples, ensuring both internal and external validation. Finally, the construction of miRNA interaction networks expanded the potential applications of these genes, laying a solid foundation for further in-depth exploration. Nonetheless, our study has limitations. First, platform heterogeneity across datasets (all Affymetrix but different versions) may introduce technical bias, though mitigated by rigorous normalization. Second, while internal and external validations were conducted, the clinical cohort size (n = 5/group) warrants expansion in multi-center studies. Third, therapeutic predictions (e.g., LPS/acetaminophen) require experimental validation in synovial models. Further *in vitro* and *in vivo* studies are necessary to confirm these findings and elucidate the underlying mechanisms.

## 5 Conclusion

In this study, we identified and validated four differentially expressed genes (CXCL8, CXCL2, DUSP5, and TNFSF11) as promising diagnostic biomarkers for osteoarthritis (OA), which collectively mediate immune regulation, inflammatory responses, and bone remodeling in OA pathogenesis. Our findings advance the understanding of synovial-bone crosstalk in OA progression and highlight actionable therapeutic targets. Future directions include experimental validation in OA animal models, pharmacological modulation studies of predicted drugs, prospective clinical trials stratifying early OA patients by synovial biomarker levels, and integration with imaging/clinical data in longitudinal cohorts to establish prognostic utility. These concrete next steps will accelerate the translation of our findings toward personalized OA management.

## Data Availability

The original contributions presented in the study are included in the article/Supplementary material, further inquiries can be directed to the corresponding authors.

## Footnotes

^1^ https://rnasysu.com/encori/index.php
